# Identification and Pathogenicity of *Fusarium* Isolated from Soybean in Poland

**DOI:** 10.3390/pathogens12091162

**Published:** 2023-09-14

**Authors:** Hanna Olszak-Przybyś, Grażyna Korbecka-Glinka, Elżbieta Patkowska

**Affiliations:** 1 Department of Plant Breeding and Biotechnology, Institute of Soil Science and Plant Cultivation-State Research, ul. Czartoryskich 8, 24-100 Puławy, Poland; gkorbecka@iung.pulawy.pl; 2 Department of Plant Protection, Faculty of Horticulture and Landscape Architecture, University of Life Sciences in Lublin, ul. Leszczyńskiego 7, 20-069 Lublin, Poland

**Keywords:** soybean, *Glycine max* (L.) Merrill, root rot, soil-borne fungi, pathogenicity, *Fusarium* species, translation elongation factor 1-alpha, RNA polymerase second largest subunit

## Abstract

Fungi belonging to the *Fusarium* genus are commonly isolated from soybean plants and seeds but not all of them are pathogenic. The aim of this study was to compare the pathogenicity among different *Fusarium* isolates obtained from soybean plants with disease symptoms originating from an experimental field located in the southeast of Poland. Nineteen fungal isolates were selected for the pathogenicity assay, including eight isolates of *F. oxysporum*, six isolates of *F. graminearum*, four isolates of *F. culmorum* and one isolate of *F. redolens*. Species identification of these isolates was carried out using microscopic methods and sequencing of two genes: translation elongation factor 1-alpha (*TEF1*) and RNA polymerase second largest subunit (*RPB2*). To our knowledge, this is the first report of *F. redolens* being isolated from soybean in Europe. The pathogenicity test was set up by fungal inoculation of healthy soybean seeds of three cultivars: Abelina, Atlanta and Mavka. Symptoms were assessed seven days after inoculation. Disease area percentage of *Fusarium* inoculated seeds was significantly higher compared to uninoculated control. Nineteen isolates differed in their aggressiveness as the median disease area percentage ranged between 5.0 and 88.0% depending on isolate. The obtained isolates of four *Fusarium* species may be used in the future screening of soybean cultivars for resistance to these pathogens.

## 1. Introduction

Soybean (*Glycine max* (L.) Merrill) is one of the most important crops used for the production of food and animal feed because of its high oil and protein content [1]. The global production of this crop amounts to 371.7 m tonnes and countries with the highest soybean production include the USA, Brazil, Argentina, China and India [2]. In Europe in 2021, the cultivation area of this crop amounted to approx. 5.5 m ha and the yield to 2.09 t/ha [2]. Soybean production in Poland has been dynamically growing over the previous number of years. In 2018, the soybean cultivation area in this country amounted to 5450 ha, but had increased to 9210 ha by 2021 [2]. The increasing cultivation area of soybean may lead to an increased risk of pathogens infecting this crop.

The cosmopolitan genus *Fusarium* includes economically important plant pathogens. Fusarium wilt or blight and seed, seedling, stem or root rots are typical diseases that cause economic losses in agriculture, horticulture and ornamental crops worldwide [3]. Moreover, some species belonging to this genus have the ability to produce mycotoxins and secondary metabolites which can contaminate agricultural products, resulting in destruction of food and feed or having a negative impact of human and animal health [4].

Diverse species of *Fusarium* infect soybean plants at almost every growth stage causing sudden death syndrome, leaf necrosis, damping-off, and root and pod rot [5,6]. Members of this genus are frequently and consistently isolated from soybean seeds and pods and they may lower seed quality and vigour by reducing germination [7,8]. However, soybean germination in the field may be affected not only by seed-borne fungi. Many *Fusarium* species are considered to be soil-borne pathogens of soybean. They may survive in plant debris left in the field after harvest for a long time and most *Fusarium* species also have the ability to produce more durable forms-chlamydospores [9]. After seeds are sown into the soil these fungi can colonize them causing the development of disease symptoms on seedlings and reducing emergence [10].

*F. oxysporum* Schltdl. is considered to be one of the most destructive soil-borne fungi and is a major cause of soybean root rot in North America. Fusarium root rot was first reported in Iowa in 1953 and *F. oxysporum* was the predominant fungus isolated from symptomatic soybean roots. Soybean root rot caused by this species has also been widely reported in China [11] and is the most frequently isolated *Fusarium* species from field grown soybean plants in Poland [12,13,14]. Moreover, *F. oxysporum* is associated with Fusarium wilt or blight and can cause a range of symptoms, including damping-off, cortical decay and vascular discoloration in soybean plants. Economic losses up to 59% resulting from wilt and 64% from root rot have been reported. It is also known that infections of seeds can reduce germination even up to 40% in the field [10].

The *F. redolens* Wollenw. (syn: *F. oxysporum* var. *redolens* (Wollenw.) W.L. Gordon) species is similar to the *F. oxysporum* species in terms of morphological characteristics. For this reason, this fungus has been long recognised as a variety of *F. oxysporum* [15] or has been included in the *F. oxysporum* species complex [16]. The development of molecular techniques has allowed for the recognition of *F. redolens* as a separate species.

*F. graminearum* Schwabe belongs to the *F. sambucinum* species complex [17,18]. This fungus is an economically important pathogen of cereal crops, but it can also be highly aggressive for soybean seeds and seedlings, leading to low emergence and abnormal development [19]. The first reports of *F. graminearum* causing soybean seed infection in the United States appeared in 1986. At present, this species is recognized as a primary pathogen of soybean, responsible for root rot, and for pre- and post-emergence damping-off in several countries on both American continents [17]. It is also isolated from soybean seeds as well as plants grown in Poland [14,20]. Moreover, *F. graminearum* has the ability to produce trichothecene mycotoxins, which significantly reduce seed germination [20,21].

*F. culmorum* (Wm. G. Sm.) Sacc., also belonging to the *F. sambucinum* species complex, is considered to be an economically important pathogen of cereals, particularly wheat and barley [22]. It causes crop production losses each year worldwide and is widespread in Europe, North Africa, Asia and Australia [23]. According to Hartman et al. [10], *F. culmorum* have been isolated from soybean, but there are not many reports of its pathogenicity for this crop. However, Zeng et al. [23] consider this pathogen to be one of the major causal agents of soybean root rot in three provinces in China. There are few reports of the detection of this species on soybean seed and plants in Poland [12,13,14,20,24] The pathogenicity of *F. culmorum* isolates is closely linked to production of mycotoxins, including the trichothecenes [25].

Not all *Fusarium* isolates obtained from field-collected plants are pathogenic, meaning that not all of them have the ability to induce disease symptoms in the plants. Moreover, they may differ in aggressiveness, which is commonly defined as the quantitative variation of pathogenicity on susceptible hosts [26]. Variability in pathogenicity and aggressiveness within *Fusarium* genus has been documented in previous studies [5]. Particularly with regard to *F. oxysporum*, it is clear that isolates may range from highly aggressive to non-pathogenic [27]. Therefore, testing the pathogenicity of the obtained *Fusarium* isolates should be an important element in identifying pathogens; it is also recommended in Koch’s postulates and recent guidelines for pathogenicity testing [28].

The aim of this study was to determinate the pathogenicity of nineteen *Fusarium* isolates obtained from symptomatic soybean plants grown in southeast Poland and compare their aggressiveness. Fungal isolates from soybean grown in this geographic region have not, so far, been subjected to similar research. Apart from *F. oxysporum* and *F. graminearum*, the isolates tested here included *F. culmorum*, which has been rarely studied in the context of pathogenicity for soybean seeds. Moreover, the pathogenicity of *F. redolens* isolates has never been tested in Europe on this crop.

## 2. Materials and Methods

### 2.1. Samples Collection and Fungi Isolation

Fungal isolates included in this study were obtained in June and July 2019 from soybean plants grown from uncoated seeds in the experimental field located in Makowisko (50°2′43″ N, 22°47′10″ E, podkarpackie voivodeship, southeast Poland) [14]. Soil and weather conditions in this experiment have been described by Jarecki et al. [29]. Collected soybean plants showed symptoms of fungal infection on the roots or shoots. These symptoms have included brown or reddish-brown-to-black lesions on the tap root or lateral roots. In addition, rotting of the terminal parts of the roots, vascular discoloration and chlorotic or necrotic lesions on the stems were observed. The sampled material was rinsed and surface-disinfected with 1.4% sodium hypochlorite solution for one minute, rinsed three times in the sterile water and air-dried on sterile tissue paper under aseptic conditions. Finally, the samples of organs with disease symptoms were cut into 1 cm long fragments and placed in Petri dishes on mineral SNA medium [9] with tetracycline hydrochloride (2.5 mg L^−1^). Petri dishes with plant fragments were incubated at room temperature in the darkness for 14 days, until fungal mycelium grew. The fungi that grew on the SNA medium were subsequently isolated by cutting small pieces of the mycelium and transferring them to separate Petri dishes with the potato dextrose agar (PDA, Difco^TM^, Sparks, MD, USA) medium. Then, pure isolates were obtained using a single-spore isolation method [30]. A suspension of conidia from mycelium was prepared in 5 mL sterile water on a sterile plastic Petri dish. Then, 5 µL of the conidial suspension was pipetted and transferred to the centre of the Petri dish containing 2% water agar medium. The conidial suspension was spread on the surface of the medium using a glass spatula and incubated for 12–14 h at 26 ± 2 °C. Thereafter, single germinating conidia were picked under the microscope and transferred onto Petri dishes with PDA medium. Successfully grown cultures obtained from these conidia constituted fungal isolates used in further experiments.

### 2.2. Morphological Identification and Characterization

The morphological characterization of fungal isolates was carried out after 14 days of incubation on PDA medium in the dark at 23 ± 2 °C. First, the culture appearance was noted for each isolate by describing the structure of mycelium and its colour on both sides of the culture. Then, several macroconidial features, including shape, size and the number of septa, were recorded from 100 spores of each tested isolate using a NICON Eclipse 80i microscope. The presence of microconidia was also noted during microscopic observations. The recorded data were compared with species descriptions in keys of taxonomic identification [9,31].

The fungal growth test was set up by transferring 5 mm diameter mycelial discs with a sterile corkborer from a 2 week fungal culture to the centre of a fresh Petri dish with PDA medium. The plates were incubated at 26 ± 2 °C in dark. The fungal growth was recorded daily for 4 days and measured from the edge of the initial inoculum to the extreme area of the mycelia using four perpendicular lines drawn on the reverse of the Petri dishes. This experiment was conducted in three replicates and the average values of the 4 measurements made along the lines marked on the Petri dishes were recorded at 24 h intervals. Then growth rate (in mm/day) was calculated and compared among the studied isolates using one way analysis of variance (ANOVA) and Tukey’s post-hoc test.

### 2.3. Molecular Identification of Species

The genomic DNA was extracted from fourteen-day-old isolates according to a modified CTAB method [32]. The extraction buffer contained: 3% *w*/*v* CTAB, 100 mM Tris-base, 20 mM EDTA, 1.4 M NaCl, pH = 8. The quality and the quantity of the total extracted DNA were evaluated using a NanoDrop2000 (Thermo Scientific, Wilmington, DE, USA). Following the recommendations of O’Donnell et al. [33], two highly informative genomic regions, translation elongation factor 1 alpha (*TEF1*) and RNA polymerase second largest subunit (*RPB2*), were selected for a sequence-based species identification of the tested *Fusarium* isolates. First, polymerase chain reaction (PCR) amplification was performed in a volume of 25 µL containing 12.5 µL of Platinum Green Hot Start PCR 2X Master Mix (Invitrogen, Vilnius, Lithuania), 0.2 µM of each of the two primers and 50 ng of DNA. PCRs for *TEF1* region were performed using primers EF1 and EF2, while *RBP2* region was amplified using primers 5f2 and 7cr [33]. The amplification was carried out using a C1000 thermal cycler (Bio-Rad, Singapore) using the following temperature conditions: 2 min at 94 °C, followed by 35 cycles at 94 °C for 30 s, 56 °C for 90 s and 68 °C for 3 min with a final extension step at 68 °C for 5 min. Subsequently, the obtained PCR products were treated with ExoSAP-IT reagent following the manufacturer’s protocol (Applied Biosystems, Vilnius, Lithuania) and then subjected to sequencing. Primers EF3 and EF22U were used to sequence the *TEF1* region and primers 5f2 and 7cr for the *RPB2* region [33]. Cycle sequencing reactions were performed using Big Dye Terminator v3.1 chemistry (Applied Biosystems, Vilnius, Lithuania) and Veriti thermal cycler (Applied Biosystems, Singapore) following the manufacturer’s recommendations. The products of sequencing reactions were purified using ethanol/EDTA precipitation and separated on a 3500 Genetic Analyzer (Applied Biosystems, Ibaraki, Japan). The obtained sequences were reviewed and edited using Sequencing Analysis software v.6.0 (Applied Biosystems, Foster City, CA, USA). Then forward and reverse sequences for each genomic region and each *Fusarium* isolate were trimmed and assembled into continuous sequences using MEGA software v.11 [34]. Subsequently, the obtained sequences were deposited in GenBank. These were also subjected to a search of highly similar sequences in the NCBI database (http://blast.ncbi.nlm.nih.gov/ accessed on 1 August 2023) by nucleotide BLAST and the FUSARIUM ID v.3.0. database on the Galaxy platform (http://usegalaxy.eu/datasets/edit, accessed on 1 August 2023) [35]. *Fusarium* species for each sequenced isolate were determined based on BLAST results with 99–100% identity (Tables S1 and S2).

### 2.4. Phylogenetic Analysis

BLAST searches revealed that the 19 *Fusarium* isolates studied here represent the following four species: *F. redolens*, *F. oxysporum*, *F. culmorum* and *F. graminearum*. Therefore, we downloaded representative *TEF1* and *RPB2* sequences from the NCBI database of these species from various geographic regions and included them in the phylogenetic analysis for comparative purposes (see also Table S3). We also added sequences of *F. solani* in order to root the dendrograms. Phylogenetic analysis was performed in MEGA software v.11. First, all sequences were aligned using CLUSTAL W. Then, a phylogenetic tree was constructed using the maximum likelihood (ML) method. Bootstrap values for maximum likelihood were 1000 replicates, with one search replicate per bootstrap replicate. Bootstrap values >50 were shown in phylogenetic trees.

### 2.5. Pathogenicity Test of Fusarium Species on Soybean Seeds

**Figure 1:**
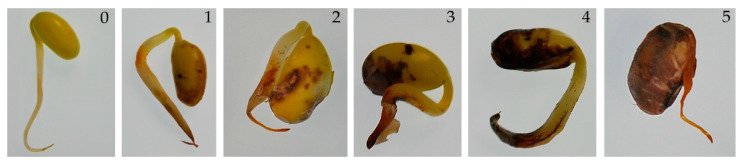
Rating scale of disease symptoms on soybean seedlings inoculated with *Fusarium* spp. Pictures of seedlings representing the following disease ratings: 0 = healthy germinated seedling with no disease symptoms (no necrosis); 1 = slight necrosis with the total diseased area up to 10%; 2 = slight-to-moderate necrosis with total diseased area between 11 and 25%; 3 = moderate necrosis with total diseased area 26–50%; 4 = extensive necrosis with total diseased area 51–75%; 5 = extensive necrosis with total diseased area over 75%.

The pathogenicity test was conducted on healthy soybean seeds of three cultivars recommended for cultivation in Poland, namely Mavka, Atlanta and Abelina. The test for each cultivar was performed in three replicates of 100 seeds each. The seed soaking method was used in the inoculation process [7]. Spore suspension was prepared from single-spore cultures grown on PDA medium in 9 cm diameter Petri dishes. Under a sterile laminar flow chamber, 14 day-old mycelium of each of the *Fusarium* isolates was transferred to a sterile glass bottle with 70 mL potato dextrose broth (PDB, Difco^TM^) medium and incubated at 150 r min^−1^ and 25 °C on an orbital shaker Innova 40R (New Brunswick Scientific, CT, USA) for 7 days. Then, the suspension was poured through a sterile sieve with 0.8 mm diameter holes in order to remove excess mycelium that had grown during the suspension shaking process. The concentration of this was assessed using a hemacytometer under a NICON Eclipse 80i microscope at 400 times magnification. Then, the concentration of the spore suspension was adjusted to 1 × 10^6^ spores per mL with a sterile PDB medium and used for inoculation. Soybean seeds used in this study were surface disinfected with 1.4% sodium hypochlorite solution (*v*/*v*) for one minute, rinsed three times in sterile distilled water and placed on sterile filter paper in a Petri dish to dry. Subsequently, disinfected seed material was dipped in the spore suspension for 5 min, drained and placed in a Petri dish with sterile tissue paper. The seeds soaked in PDB medium without spores constituted negative controls. Then, Petri dishes with inoculated and control seeds were kept under controlled conditions (25 ± 2 °C in the dark with 70% humidity) in Micro Clima plant growth chambers (Snijders Labs, Tilburg, The Netherlands). During incubation, filter papers in Petri dishes were moistened with sterilized water to prevent germinating seeds from drying out. Seven days after inoculation, abundant mycelium and seed coats were removed from the seeds/seedlings to allow a precise assessment of disease symptoms visible also on cotyledons and radicle, which were mostly brown, black or rotten areas of these organs (necrosis). The disease symptoms of the seeds/seedlings were recorded according to a 0–5 arbitrary scale modified after Zang et al. [36] in which: 0 = healthy germinated seedling with no disease symptoms (no necrosis); 1 = slight necrosis with the total diseased area up to 10%; 2 = slight-to-moderate necrosis with total diseased area between 11 and 25%; 3 = moderate necrosis with total diseased area 26–50%; 4 = extensive necrosis with total diseased area 51–75%; and 5 = extensive necrosis with total diseased area over 75% or complete decay of the seed (Figure 1).

In addition, for each individual soybean seedling, the length of its primary root (radicle) was measured. Significance level was set at α = 0.05. Then, a representative sample of inoculated seeds/seedlings was placed on a PDA medium in order to obtain fungal cultures. This was undertaken in order to confirm infection by the fungus used for inoculation, following Koch’s postulates. The obtained fungal cultures were subjected to DNA extraction, PCR amplification and sequencing of the *TEF1* region as described in Section 2.3. The sequences of these cultures were identical to sequences of the original *Fusarium* isolates used for inoculation.

### 2.6. Statistical Analysis

All data were processed using Microsoft Office Excel, whereas statistical analyses were carried out using software Statistica version 13.3 (Tibco Software, Palo Alto, CA, USA). Data on disease symptoms ratings, germination percentage and radicle length were obtained from 900 seeds for each isolate (3 × 100 seeds for each cultivar). In turn, measurements of macroconidia structures were carried out for 100 items of each isolate. The significance of the observed differences among tested isolates in the growth rate and macroconidia measurements were tested by means of one-way analysis of variance (ANOVA) and a subsequent post-hoc Tukey’s test. Disease ratings were replaced by a mid-point of the range of percentage of the diseased area specified for each rating. Then, these percentage data were subjected to a non-parametric Kruskal–Wallis test to compare the aggressiveness of all of the tested *Fusarium* isolates. The same test was used to analyse statistical differences in soybean germination percentage and radicle length among treatments with different isolates.

## 3. Results

### 3.1. Morphological and Molecular Identification of Fungal Isolates

Based on cultural and morphological features such as growth rate, length and width of macroconidia, basal cell shape, number of septa in macroconidia and presence or absence of microconidia in the culture, nineteen tested isolates were tentatively classified into three different groups. The first distinct group included isolate nos. 1–9, while the second and the third groups contained isolate nos. 10–13 and nos. 14–19, respectively (Table 1).

Cultures of isolates belonging to the first group grown on PDA medium were generally characterized by an abundant white-to-pale-purple cottony mycelium and a dark purple under the surface. The average growth rate of these cultures ranged between 4.49 and 6.27 mm/day. Macroconidia were relatively slender, 30.35–39.42 µm long on average, thin walled and had 2–5 septa. Their average width ranged between 3.03 and 4.42 µm and deviated significantly from the macroconidia width of isolates in the other two groups, with the exception of one isolate (Table 1). Microconidia were observed only in the cultures of isolate nos. 1–9; these had an oval or elliptical shape with no septa inside.

The second group included isolates with cultures characterized by a dark mycelium colour from pastel red to deep red. Of all the tested isolates, the lowest average growth rate was recorded for cultures of isolate nos. 10–13. It ranged between 2.36 and 3.77 mm/day. The observed macroconidia had no foot and were relatively short and thick, with average length and width varying between 27.00 and 32.03 µm and between 5.29 and 5.68 µm, respectively. Microconidia were not observed in cultures of these isolates.

Cultures of isolates from the third group were coloured yellowish brown to reddish brown, sometimes with a central mass in red, and with the reverse red. Their average growth rate was rather high, ranging between 5.32 and 7.71 mm/day. Of all the tested isolates, the fastest growing belong to this group. Macroconidia were slender, thick-walled and significantly longer compared with the macroconidia of isolates from other two groups (Table 1). Their average length varied between 52.08 and 53.82 µm. Macroconidia width reached intermediate values and ranged between 4.80 and 5.10 µm. These spores had 2–7 septa and their basal cell had a clearly visible foot. Microconidia were not observed in cultures of these isolates either.

Final assignment of nineteen isolates of the *Fusarium* species was performed based on the results on BLAST analysis of the *TEF1* and *RPB2* sequences (GeneBank accession numbers: OP985466—OP985484 and OR248153—OR248171 for *TEF1* and *RPB2*, respectively) against NCBI and FUSARIUM ID v3.0 databases. BLAST results reveal a high level of identity (99–100%) between the tested sequences and the sequences deposited in these two databases. Based on the results with the highest sequence similarity, isolate nos. 2–9 were identified as *F. oxysporum*, isolate nos. 10–14 as *F. culmorum* and isolate nos. 15–19 as *F. graminearum* (Tables S1 and S2). Isolate no. 1 was recognised as another species, *F. redolens*, although it could not be distinguished from the other isolates in the first group based on the results of mycological analysis. Culture growth rate and the macroconidia length and width of the *F. redolens* isolate fit into the ranges recorded for *F. oxysporum* isolate nos. 2–9 (Table 1); therefore, these two species were distinguished only on the basis of the *TEF1* and *RPB2* sequences.

**Figure 2:**
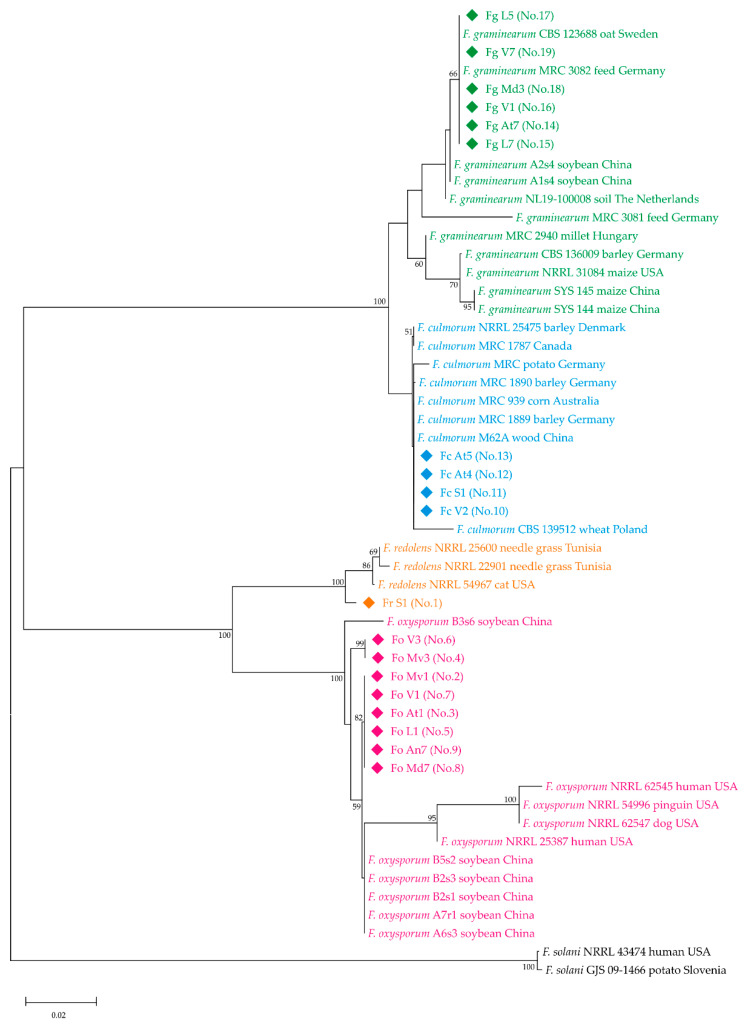
Phylogenetic tree inferred by maximum likelihood (ML) analysis of the concatenated, partial sequences of translation elongation factor 1-alpha (*TEF1*) and RNA polymerase second largest subunit *(RPB2*). The ML analysis was based on the sequences of 19 *Fusarium* isolates characterised in this study (indicated by diamonds next to the isolate names) and four *Fusarium* species obtained from GenBank (for which the following information is provided: species name, isolate name, isolation source and a country of origin; GenBank accession numbers of these records are provided in Table S3). The tree was rooted on *F. solani*. The numbers above the nodes represent ML bootstrap support (indicated if higher than 50%) based on 1000 replications of the data implemented in MEGA v.11.

Phylogenetic analysis confirmed assignment of the studied isolates to the four above-mentioned *Fusarium* species. Separate analyses performed for the *TEF1* and *RPB2* regions yielded consistent results (Figure 2). Both ML trees formed four well-supported clades. Clade 1 included six isolates (14–19) and NCBI records of *F. graminearum*. Clade 2 included four isolates (10–13) and NCBI records of *F. culmorum*. Clade 3 contained one isolate (1) and NCBI records of *F. redolens* and clade 4 consisted of eight isolates (2–9) and NCBI records of *F. oxysporum*.

### 3.2. Pathogenicity of the Obtained Isolates

Seven days after inoculation, soybean seeds were covered with mycelium and necrotic symptoms (brown, black or rotten tissues) were visible on radicles and cotyledons. In contrast, uninoculated control seeds remained asymptomatic (Figure S1). Disease area percentage of *Fusarium*-inoculated seeds was significantly higher compared with that of the control (Table 2). Therefore, all tested isolates were able to infect soybean seeds and they were all pathogenic for this crop. Nineteen isolates differed with aggressiveness; median disease area percentage ranged between 5.0 and 88.0%, depending on the isolate, and there were statistically significant differences among them (Table 2). The most aggressive isolates for all three soybean cultivars were *F. oxysporum* isolate nos. 6 and 7 because the highest proportion of seeds inoculated with these isolates showed the most severe symptoms (diseased area > 75% or complete decay of the seed; Figure S2). In contrast, *F. culmorum* isolate no. 13 was the least aggressive as the median diseased area percentage equalled 5.0% (Table 2). 

Seeds of the Abelina cultivar suffered the most from infection because 12 out of all tested isolates caused severe symptoms (diseased area > 75% or complete decay of the seed) on over half of the inoculated seeds (Figure S2), and is also manifested in the way in which the median diseased area reached 88.0% (Table 2). In contrast, such severe effects of the disease were observed after inoculation with two and three isolates on the Atlanta and Mavka cultivars, respectively.

Generally, inoculation with tested isolates lowered germination percentage compared with the control as it ranged between 12 and 83%, 58 and 96% and 24 and 93%, for cultivars Abelina, Atlanta and Mavka, respectively; however, it was significantly lower compared with the control only in the case of inoculation with the most aggressive isolates (nos. 6 and 7). Infection led to growth inhibition of the primary root (radicle), as the median of its length was significantly lower compared with the uninoculated control in the cases of most isolates for the Abelina cultivar. For the other two cultivars, this effect was observed for most *F. oxysporum* isolates.

## 4. Discussion

Germination in the field is a vulnerable stage of soybean development in which seeds and seedlings are exposed to soil-borne pathogenic fungi, especially in the conditions of high humidity and low temperature. Hartman et al. [10] have listed seven *Fusarium* species associated with soybean root rot on both American continents. Here, we tested the pathogenicity of 19 isolates previously obtained from plants with root rot symptoms from a field in southeast Poland. Pathogenicity tests of *Fusarium* fungi must be preceded with species identification because many members of this genus may be responsible for similar symptoms.

The accurate identification of fungal pathogens often requires the adoption of a polyphasic approach based on morphology, ecology and molecular methods [28]. The sequencing of one or two genes has become a standard method of confirming species assignment within the *Fusarium* genus [37]. O’Donnell et al. [33] have recommended sequencing regions which are universally informative within *Fusarium*: *TEF1* and RNA polymerase largest subunit (*RPB1*) and/or *RPB2*. In the case of our study, the *TEF1* and *RPB2* sequences proved to be sufficient for the unambiguous assignment of the nineteen isolates to four *Fusarium* species. Many recent studies have used the same two regions for *Fusarium* species identification [7,8,38,39,40,41,42,43]. Our research has confirmed that sequencing is essential for distinguishing *Fusarium* species. The preliminary analysis of the morphological characteristics, such as culture appearance, growth rates and macroconidia measurements, did not show statistically significant differences between isolate no. 1 and all of the *F. oxysporum* isolates (nos. 2–9). Only sequencing *TEF1* and *RPB2* genes revealed that isolate no. 1 belongs to a different species, *F. redolens*.

To our knowledge, this is the first time that *F. redolens* has been isolated from soybean in Europe, although there is a possibility that it was identified as *F. oxysporum* in earlier studies. The reason for this is that the taxonomic status of *F. redolens* changed a number of times over the 20th century and some authors included it into their concept of *F. oxysporum* or considered it to be a variety of *F. oxysporum* [9]. Because molecular techniques allow for a clear distinction of both species, numerous studies have described the isolation of *F. redolens* from a wide range of crops, including chickpea, pea, lentil, asparagus, corn, wheat, potato and sugar beet [44,45,46,47,48,49]. However, currently, all reports of detecting it on soybean originate from North America [50,51].

The nineteen isolates included in this study represent four *Fusarium* species: *F. redolens*, *F. oxysporum*, *F. culmorum* and *F. graminearum*. All of these isolates appeared to be pathogenic to soybean seeds, although two *F. oxysporum* isolates (nos. 6 and 7) were clearly the most aggressive for all three cultivars compared with other isolates. There are many reports on the pathogenicity of *Fusarium* isolates which are based on soil-free experiments in which soybean seeds are germinated, grown in Petri dishes or paper towels. Their outcome is often similar to our results: all tested isolates were pathogenic but differed in their aggressiveness [8,52,53]. Conditions in such pathogenicity tests (high humidity and soil-free environment) may favour disease development, hence their result may be overestimated. Therefore, laboratory pathogenicity tests should be treated as preliminary studies and ideally their results should be confirmed in greenhouse or field experiments, as suggested, for example, by Hartman et al. [21].

*F. oxysporum* is a ubiquitous species which is frequently isolated from crops, including soybean [36,44]. This species is generally characterised by a high genetic diversity which also affects its aggressiveness. Variation in the severity of symptoms triggered by different *F. oxysporum* isolates is frequently reported. Diaz Arias et al. [5] compared the aggressiveness of 14 isolates of this species obtained from the roots of soybean grown in Iowa (USA). All isolates had detrimental effects on the growth of experimental soybean plants; however, only for one of these did root rot severity differ statistically significantly from the non-inoculated control. In another study, Ellis et al. [52] characterized over 100 isolates of *F. oxysporum* collected from soybean roots and seedlings in the USA and proved their genetic diversity. Based on laboratory pathogenicity assay, they also categorised these isolates into highly, moderately and weakly aggressive. The most aggressive isolates induced clear symptoms of wilt, damping-off and root rot on soybean seedlings.

*F. graminearum* causes root rot and seedling disease of soybean with symptoms including elongate lesions on roots and shoots which are first light brown then become necrotic, leading to wilting and the death of the plants [10]. In comparative studies on the pathogenicity of *Fusarium* species on soybean, *F. graminearum* often stands out as the species which is among the most aggressive for this crop. For example, Diaz Arias et al. [5] compared, in a greenhouse experiment, the aggressiveness of nine *Fusarium* species and found that *F. graminearum* strains caused the most severe root rot symptoms. A similar conclusion was made in two other studies comparing the aggressiveness of *Fusarium* isolates for soybean seed and seedlings [36,54]. Variation in aggressiveness among *F. graminearum* isolates for this crop has also been reported. For example, Parikh et al. [44] have demonstrated significant differences in disease severity recorded on soybean seedlings after inoculation with eight isolates of this species. A similar result was presented by Bonacci et al. [53], based on pathogenicity assay with ten *F. graminearum* isolates.

The other two species included in our research, *F. culmorum* and *F. redolens*, have been rarely studied in the context of their pathogenicity on soybean seedlings. *F. culmorum* was found to be pathogenic for soybean in Canada [50,55]. Although, Zeng et al. [23] describe this species as one of the major causal agents of soybean root rot in three provinces in China. To our knowledge, data on *F. redolens* pathogenicity to soybean is equally limited; it has been reported in only two studies [50,51].

The pathogenicity of *Fusarium* spp. is determined by a wide range of factors, including the genes responsible for signal transduction, detoxifying antifungal compounds produced by plants, metabolic enzymes and cell wall-degrading enzymes [56,57]. Genes responsible for the pathogenicity of the *Fusarium* species, located on accessory chromosomes, can be horizontally transferred from one strain to another [58,59]. Therefore, isolates of different *Fusarium* species obtained from the same location may share these pathogenicity factors and have a similar impact on plants.

Many *Fusarium* species have the ability to produce mycotoxins, such as trichothecenes, which can be toxic to plants [60]. Evidence that these compounds contribute to the pathogenicity of fungi has been provided, for example, by Hestbjerg et al. [61], who found a significant positive correlation between the concentration of deoxynivalenol (belonging to trichothecenes), and disease index recorded for barley seedlings inoculated with 70 *F. culmorum* isolates. Our isolates belonging to *F. culmorum* and *F. graminearum* species may be subject to similar research in the future.

In our pathogenicity tests, we found that inoculated seeds of Abelina cultivar showed the strongest reduction of germination percentage and the strongest radicle growth inhibition compared with the Atlanta and Mavka cultivars. The information that the Abelina cultivar is more susceptible to *Fusarium* infection may be useful for planning future testing of pathogenicity of fungal isolates representing this genus. Nevertheless, it is important to note that none of the tested three soybean cultivars was resistant to *Fusarium* infection. Finding sources of resistance to these pathogens would require large-scale pathogenicity tests using a high number of soybean cultivars [9].

## Figures and Tables

**Table 1:** Characteristics of nineteen *Fusarium* isolates, including description of culture appearance and its average growth rate (±standard deviation) based on measurements taken after 4 days of growth. Macroconidia measurements include their average (±standard deviation), length, width and the number of septa. Table also contains information on the presence (+) or absence (−) of foot on macroconidia and of microconidia in the culture. Dashed lines separate the three isolate groups distinguished during mycological analysis.

Isolate Number	Isolate Name #	Culture Appearance	Growth Rate (mm/day)	Macroconidia				Microconidia
				Length (µm)	Width (µm)	Septa Number	Foot	
1	Fr_S1	White, flocky, reverse white	6.08 ± 0.19 ^efg^*	35.49 ± 8.83 ^def^*	3.57 ± 0.56 ^bcd^*	2–3	+	+
2	Fo_Mv1	Pale purple, reverse light purple in the centre	5.46 ± 0.04 ^def^	34.06 ± 9.94 ^cde^	4.42 ± 0.71 ^e^	2–5	+	+
3	Fo_At1	White, flocky, reverse white	5.72 ± 0.33 ^def^	30.35 ± 8.80 ^abc^	3.88 ± 0.47 ^cd^	2–4	+	+
4	Fo_Mv3	Dark purple, white margins, reverse purple	5.55 ± 0.07 ^def^	38.80 ± 8.86 ^ef^	3.03 ± 0.82 ^a^	2–5	+	+
5	Fo_L1	Pale purple, flocky, reverse purple	4.95 ± 0.02 ^cde^	36.28 ± 8.69 ^def^	3.31 ± 0.89 ^ab^	2–5	+	+
6	Fo_V3	Light purple and flocky, reverse pale purple	6.04 ± 0.21 ^defg^	38.34 ± 8.41 ^ef^	3.67 ± 0.72 ^bcd^	2–5	+	+
7	Fo_V1	White, reverse purple in the centre and white at the margins	4.79 ± 0.67 ^cd^	39.26 ± 5.10 ^f^	3.48 ± 0.81 ^bc^	2–5	+	+
8	Fo_Md7	Light purple, fluffy and flocky, reverse pale purple	5.83 ± 0.02 ^def^	35.96 ± 8.52 ^def^	3.89 ± 0.70 ^d^	2–5	+	+
9	Fo_An7	White with fluffy growth, reverse white and flocky	6.27 ± 0.25 ^fg^	39.42 ± 6.20 ^f^	3.94 ± 0.77 ^d^	2–5	+	+
10	Fc_V2	Deep red, reverse dark red	3.45 ± 0.99 ^ab^	28.15 ± 6.34 ^ab^	5.68 ± 1.01 ^i^	2–5	−	−
11	Fc_S1	Pastel red, orange-yellow margins, reverse pale red	2.36 ± 0.44 ^a^	27.00 ± 3.36 ^a^	5.64 ± 0.85 ^i^	2–4	−	−
12	Fc_At4	Dark red, reverse red with beige margins	3.77 ± 1.08 ^bc^	32.03 ± 5.10 ^bcd^	5.47 ± 0.75 ^hi^	2–4	−	−
13	Fc_At5	Deep red, light red margins, reverse pastel red	2.54 ± 0.28 ^ab^	29.01 ± 4.69 ^ab^	5.29 ± 0.81 ^ghi^	2–4	−	−
14	Fg_At7	Pastel red to light yellow, reverse red	6.56 ± 0.22 ^fgh^	53.11 ± 14.04 ^g^	4.80 ± 1.02 ^ef^	2–7	+	−
15	Fg_L7	White to pale pink, reverse strongly red in the centre	7.30 ± 0.08 ^gh^	53.82 ± 15.27 ^g^	4.98 ± 0.94 ^fg^	3–7	+	−
16	Fg_V1	Pastel red to light yellow, reverse red	5.73 ± 0.20 ^def^	52.89 ± 13.08 ^g^	5.02 ± 0.87 ^fg^	3–7	+	−
17	Fg_L5	White yellowish, reverse yellowish brown	7.71 ± 0.08 ^h^	53.35 ± 14.03 ^g^	4.99 ± 0.90 ^fg^	3–7	+	−
18	Fg_Md3	Light yellow to greyish red, reverse orange and yellow	5.96 ± 0.18 ^def^	52.08 ± 14.31 ^g^	4.86 ± 0.99 ^f^	3–7	+	−
19	Fg_V7	White to pale pink, fluffy, reverse white and flocky	5.32 ± 0.16 ^def^	53.38 ± 12.33 ^g^	5.10 ± 0.78 ^fgh^	3–7	+	−

# Isolate names include abbreviated fungal species name (as determined based on BLAST of *TEF1* and *RBP2* sequences: Fr, *F. redolens*; Fo, *F. oxysporum*; Fc, *F. culmorum*; Fg, *F. graminearum*. See Tables S1 and S2). They also contain abbreviated soybean cultivar (S, Smuglyanka; Mv, Mavka; At, Atlanta; L, Lajma; V, Violetta; Md, Madlen; An, Annushka) and a plant number from which the isolate was obtained. * Different lowercase letters in the same column indicate a significant difference (*p* < 0.05), according to Tukey’s post-hoc test.

**Table 2:** Effects of inoculation with 19 *Fusarium* isolates on diseased area percentage, radicle length and germination percentage of 3 soybean cultivars. Table contains median values for each variable.

Isolate No.	cv. Abelina			cv. Atlanta			cv. Mavka		
	DA%	RL (mm)	G%	DA%	RL (mm)	G%	DA%	RL (mm)	G%
1	88.00 ^bc^*	9.00 ^def^*	61.00 ^ab^*	63.00 ^cde^*	22.00 ^cdefg^*	91.00 ^ab^*	38.00 ^efg^*	20.00 ^bc^*	89.00 ^ab^*
2	88.00 ^bc^	9.50 ^bcd^	57.00 ^ab^	63.00 ^cd^	40.00 ^ab^	82.00 ^ab^	63.00 ^bcd^	20.00 ^bc^	87.00 ^ab^
3	38.00 ^de^	5.00 ^defghi^	63.00 ^ab^	38.00 ^gh^	10.00 ^jk^	79.00 ^ab^	38.00 ^g^	9.50 ^ef^	86.00 ^ab^
4	18.00 ^ef^	5.00 ^ghi^	69.00 ^ab^	18.00 ^h^	10.00 ^jk^	83.00 ^ab^	5.00 ^h^	6.00 ^f^	88.00 ^ab^
5	88.00 ^bc^	4.00 ^fghi^	54.00 ^ab^	38.00 ^gh^	15.00 ^ij^	84.00 ^ab^	38.00 ^defg^	10.00 ^ef^	83.00 ^ab^
6	88.00 ^a^	0.00 ^j^	23.00 ^b^	88.00 ^a^	5.00 ^k^	63.00 ^b^	88.00 ^a^	0.00 ^g^	42.00 ^b^
7	88.00 ^a^	0.00 ^j^	12.00 ^b^	88.00 ^b^	11.50 ^hij^	58.00 ^b^	88.00 ^a^	0.00 ^g^	24.00 ^b^
8	88.00 ^bc^	7.00 ^defg^	60.00 ^ab^	38.00 ^defg^	20.50 ^fgh^	82.00 ^ab^	38.00 ^g^	20.00 ^bc^	83.00 ^ab^
9	63.00 ^cd^	10.00 ^cde^	64.00 ^ab^	38.00 ^fgh^	20.00 ^ghi^	85.00 ^ab^	38.00 ^g^	20.00 ^bc^	87.00 ^ab^
10	88.00 ^bc^	10.00 ^cde^	56.00 ^ab^	38.00 ^defg^	21.00 ^efg^	84.00 ^ab^	63.00 ^bcde^	19.00 ^cde^	84.00 ^ab^
11	88.00 ^bc^	10.00 ^cde^	60.00 ^ab^	63.00 ^defg^	20.00 ^fghi^	87.00 ^ab^	63.00 ^bc^	19.00 ^bcd^	83.00 ^ab^
12	38.00 ^de^	10.00 ^bc^	78.00 ^ab^	38.00 ^efgh^	35.50 ^ab^	91.00 ^ab^	38.00 ^fg^	23.50 ^b^	84.00 ^ab^
13	5.00 ^f^	10.00 ^b^	83.00 ^ab^	5.00 ^i^	35.00 ^a^	96.00 ^ab^	5.00 ^h^	35.00 ^a^	93.00 ^ab^
14	88.00 ^b^	5.00 ^efghi^	55.00 ^ab^	63.00 ^bc^	35.00 ^ab^	98.00 ^abc^	38.00 ^cdefg^	21.00 ^bc^	88.00 ^ab^
15	88.00 ^bc^	0.00 ^hi^	44.00 ^ab^	63.00 ^defg^	30.00 ^bcdef^	85.00 ^ab^	88.00 ^b^	10.00 ^def^	76.00 ^ab^
16	18.00 ^ef^	20.00 ^ab^	71.00 ^ab^	18.00 ^h^	32.00 ^abcd^	87.00 ^ab^	18.00 ^h^	34.00 ^a^	90.00 ^ab^
17	38.00 ^de^	10.00 ^cde^	63.00 ^ab^	38.00 ^defg^	39.00 ^abc^	81.00 ^ab^	50.50 ^cdefg^	29.00 ^ab^	84.00 ^ab^
18	88.00 ^bc^	5.00 ^defgh^	52.00 ^ab^	38.00 ^defg^	39.50 ^ab^	88.00 ^ab^	63.00 ^bcdef^	16.00 ^cde^	74.00 ^ab^
19	88.00 ^b^	0.00 ^i^	31.00 ^ab^	63.00 ^def^	29.00 ^defg^	65.00 ^ab^	63.00 ^bcdef^	30.00 ^ab^	77.00 ^ab^
control	0.00 ^g^	20.00 ^a^	98.00 ^a^	0.00 ^j^	25.00 ^abcde^	100.00 ^a^	0.00 ^i^	20.00 ^bc^	100.00 ^a^

Isolate numbers as in Table 1. * The presence of different lowercase numbers in the same column indicates a significant difference (*p* < 0.05) according to pairwise significance tests performed after Kruskal–Wallis test. Abbreviations: DA%, disease area percentage; RL, radicle length; G%, germination percentage.

## Data Availability

Not applicable.

## Supplementary Materials

The following supporting information can be downloaded at: https://www.mdpi.com/article/10.3390/pathogens12091162/s1, Figure S1: Soybean seeds with disease symptoms on radicles and cotyledons after inoculation with *Fusarium* spp. isolates. Figure S2: Percentage of seeds assigned to a 0–5 disease rating after inoculation with 19 *Fusarium* isolates calculated for three soybean cultivars. Table S1: BLASTn results for translation elongation factor (*TEF1*) gene of the 19 *Fusarium* isolates. Table S2: BLASTn results for RNA polymerase second largest subunit (*RPB2*) gene sequences of the 19 *Fusarium* isolates. Table S3: Details on the origin of the reference *Fusarium* spp. isolates (including the species, isolate name, source and country of isolation) and the GenBank accession numbers of their *TEF1* and *RPB2* sequences included in phylogenetic analysis.
