# Supplementary material for: Identification and Pathogenicity of Fusarium Isolated from Soybean in Poland
Source: Pathogens. 2023 Sep 14;12(9):1162. doi: 10.3390/pathogens12091162 (PMC10537759; doi:10.3390/pathogens12091162)
Supplement: Supplementary file 1 [file pathogens-12-01162-s001.zip › Figure S1-S2.pdf]

pathogens

Supplementary figures

Identification and Pathogenicity of *Fusarium* Isolated from Soybean in Poland

Hanna Olszak-Przybyś [1,*], Grażyna Korbecka-Glinka [1] and Elżbieta Patkowska [2,*]

[1] Department of Plant Breeding and Biotechnology, Institute of Soil Science and Plant Cultivation-State Research, ul. Czartoryskich 8, 24-100 Puławy, Poland; gkorbecka@iung.pulawy.pl
[2] Department of Plant Protection, Faculty of Horticulture and Landscape Architecture, University of Life Sciences in Lublin, ul. Leszczyńskiego 7, 20-069 Lublin, Poland
* Correspondence: holszak@iung.pulawy.pl (H.O.-P.); elzbieta.patkowska@up.lublin.pl (E.P.)

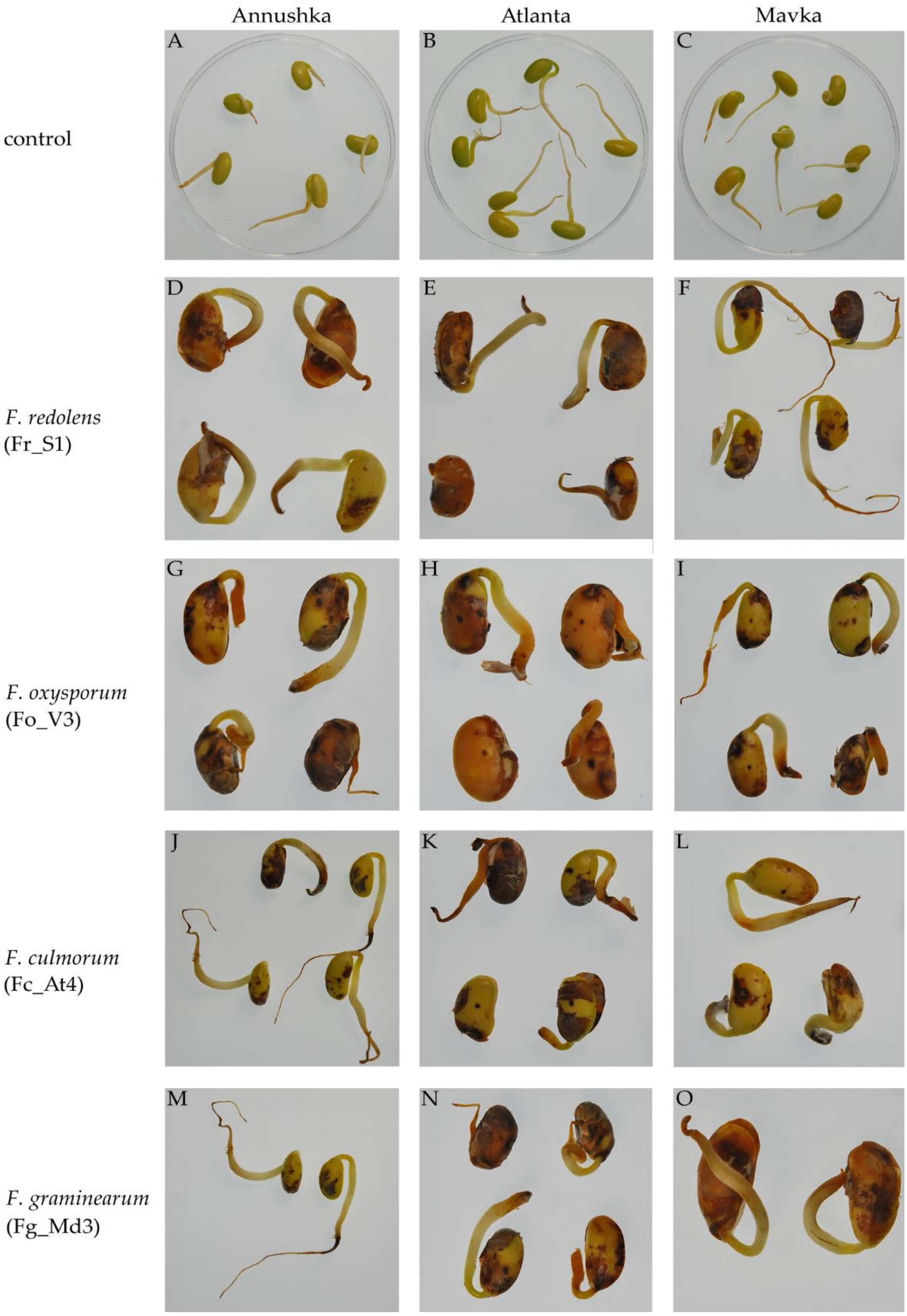

Figure S1. Exemplary pictures of seeds of three soybean cultivars (Annushka, Atlanta and Mavka) with disease symptoms on radicles and cotyledons after inoculation with *F. redolens* (D-F), *F. oxysporum* (G-I), *F. culmorum* (J-L) and *F. gramineraum* (M-O) isolates. Control comprised of seeds soaked with medium without fungal spores (A-C).

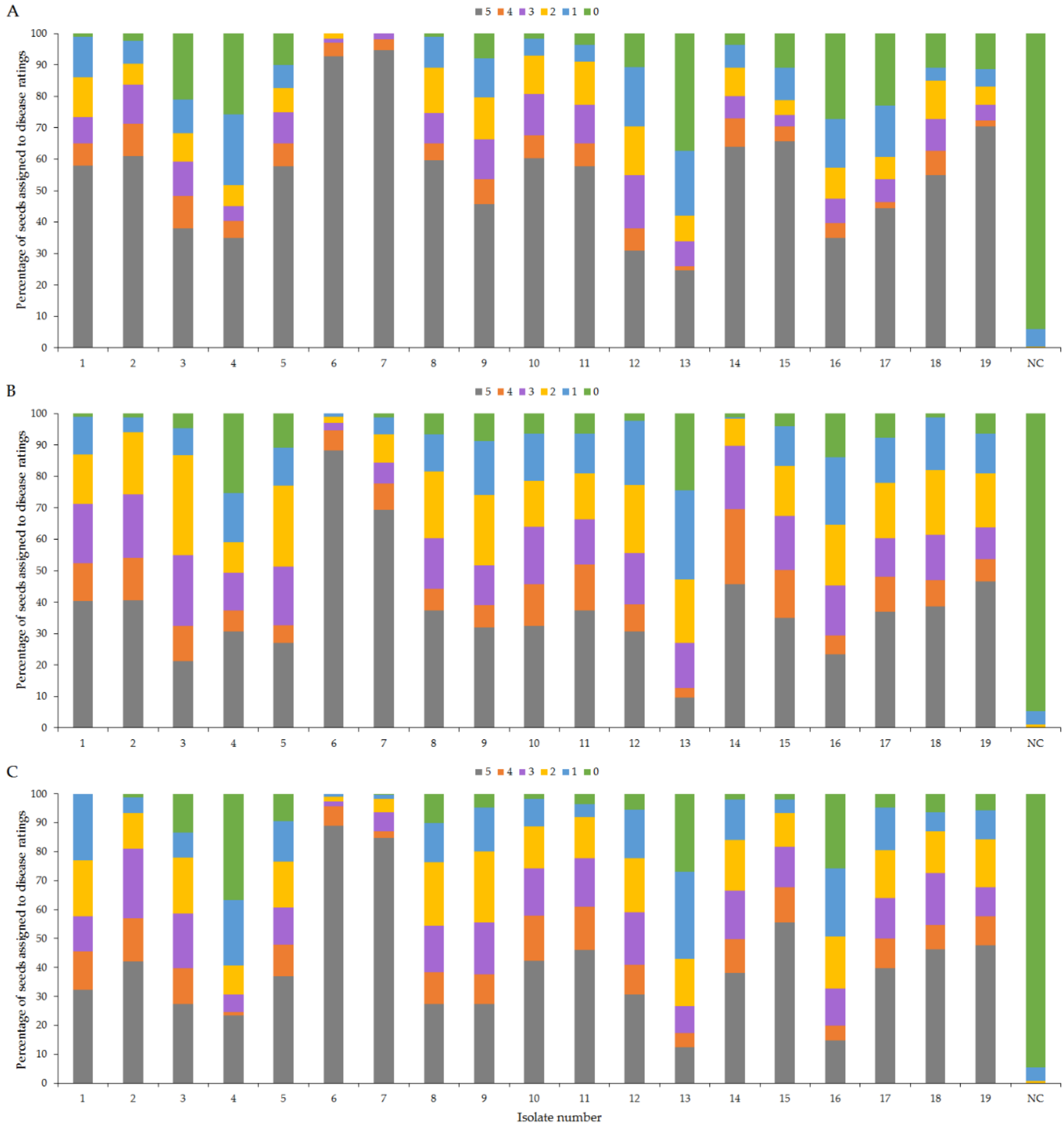

Figure S2. Percentage of seeds assigned to 0-5 disease ratings after inoculation with 19 *Fusarium* isolates (isolate numbers as in Table 1) calculated for three soybean cultivars: Abelina (**A**), Atlanta (**B**) and Mavka (**C**). NC-non-inoculated control. Disease ratings were assigned based on the observed diseased area of the seed/seedlings as follows: 0= healthy germinated seedling with no disease symptoms (no necrosis); 1= slight necrosis with the total diseased area up to 10%; 2= slight to moderate necrosis with total diseased area between 11-25%; 3= moderate necrosis with total diseased area 26-50%; 4= extensive necrosis with total diseased area 51-75%; 5= extensive necrosis with total diseased area over 75%.
