# Supplementary material for: Identification and Pathogenicity of Fusarium Isolated from Soybean in Poland
Source: Pathogens. 2023 Sep 14;12(9):1162. doi: 10.3390/pathogens12091162 (PMC10537759; doi:10.3390/pathogens12091162)
Supplement: Supplementary file 1 [file pathogens-12-01162-s001.zip › Tables S1-S3.pdf]

pathogens

Supplementary tables

Identification and Pathogenicity of *Fusarium* Isolated from Soybean in Poland

Hanna Olszak-Przybyś [1,*], Grażyna Korbecka-Glinka [1] and Elżbieta Patkowska [2,*]

[1] Department of Plant Breeding and Biotechnology, Institute of Soil Science and Plant Cultivation-State Research, ul. Czartoryskich 8, 24-100 Puławy, Poland; gkorbecka@iung.pulawy.pl
[2] Department of Plant Protection, Faculty of Horticulture and Landscape Architecture, University of Life Sciences in Lublin, ul. Leszczyńskiego 7, 20-069 Lublin, Poland
* Correspondence: holszak@iung.pulawy.pl (H.O.-P.); elzbieta.patkowska@up.lublin.pl (E.P.)

pathogens MDPI

Table S1. BLASTn results for translation elongation factor (*TEF1)* gene sequences of each 19 *Fusarium* isolates. For each compared sequence top three highly similar (with the highest percentage of identity) results are provided.

Accession number	Isolate name	Isolate number	Top three results from BLASTn				
			Accession number	Accession description	Query cover	Percent identity	E-value
OP985466	Fr_S1	1	KP964872.1	*Fusarium redolens* isolate NL96, *TEF1* gene, partial coding sequence	100%	100.00%	0.0
			KP674190.1	*Fusarium redolens* isolate M119, *TEF1* gene, partial coding sequences	100%	100.00%	0.0
			MW831872.1	*Fusarium redolens* strain BMU 03123, *TEF1* gene, partial coding sequence	100%	100.00%	0.0
OP985467	Fo_Mv1	2	KP400729.1	*Fusarium oxysporum* isolate Ch13, *TEF1* gene, partial coding sequence	100%	100.00%	0.0
			KP400726.1	*Fusarium oxysporum* isolate Ch6, *TEF1* gene, partial coding sequence	100%	100.00%	0.0
			KP400725.1	*Fusarium oxysporum* isolate Ch5A, *TEF1* gene, partial coding sequence	100%	100.00%	0.0
OP985468	Fo_At1	3	KP400729.1	*Fusarium oxysporum* isolate Ch13, *TEF1* gene, partial coding sequence	100%	99.86%	0.0
			KP400726.1	*Fusarium oxysporum* isolate Ch6, *TEF1* gene, partial coding sequence	100%	99.86%	0.0
			KP400725.1	*Fusarium oxysporum* isolate Ch5A, *TEF1* gene, partial coding sequence	100%	99.86%	0.0
OP985469	Fo_Mv3	4	KP964857.1	*Fusarium oxysporum* isolate A13, *TEF1* gene, partial coding sequence	100%	100.00%	0.0
			OM212398.1	*Fusarium oxysporum* strain VT18, *TEF1* gene, partial coding sequence	100%	100.00%	0.0
			OM212397.1	*Fusarium oxysporum* strain VT9, *TEF1* gene, partial coding sequence	100%	100.00%	0.0
OP985470	Fo_L1	5	KP400729.1	*Fusarium oxysporum* isolate Ch13, *TEF1* gene, partial coding sequence	100%	99.86%	0.0
			KP400726.1	*Fusarium oxysporum* isolate Ch6, *TEF1* gene, partial coding sequence	100%	99.86%	0.0
			KP400725.1	*Fusarium oxysporum* isolate Ch5A, *TEF1* gene, partial coding sequence	100%	99.86%	0.0
OP985471	Fo_V3	6	KP964857.1	*Fusarium oxysporum* isolate A13, *TEF1* gene, partial coding sequence	100%	99.86%	0.0
			OM212398.1	*Fusarium oxysporum* strain VT18, *TEF1* gene, partial coding sequence	100%	99.86%	0.0
			OM212397.1	*Fusarium oxysporum* strain VT9, *TEF1* gene, partial coding sequence	100%	99.86%	0.0

OP985472	Fo_V1	7	KP400729.1	*Fusarium oxysporum* isolate Ch13, *TEF1* gene, partial coding sequence	100%	100.00%	0.0
			KP400726.1	*Fusarium oxysporum* isolate Ch6, *TEF1* gene, partial coding sequence	100%	100.00%	0.0
			KP400725.1	*Fusarium oxysporum* isolate Ch5A, *TEF1* gene, partial coding sequence	100%	100.00%	0.0
OP985473	Fo_Md7	8	MT630338.1	*Fusarium oxysporum* isolate PG316, *TEF1* gene, partial coding sequence	100%	99.86%	0.0
			MT630336.1	*Fusarium oxysporum* isolate PG247, *TEF1* gene, partial coding sequence	100%	99.86%	0.0
			MT630332.1	*Fusarium oxysporum* isolate PG108, *TEF1* gene, partial coding sequence	100%	99.86%	0.0
OP985474	Fo_An7	9	KP400729.1	*Fusarium oxysporum* isolate Ch13, *TEF1* gene, partial coding sequence	100%	99.86%	0.0
			KP400726.1	*Fusarium oxysporum* isolate Ch6, *TEF1* gene, partial coding sequence	100%	99.86%	0.0
			KP400725.1	*Fusarium oxysporum* isolate Ch5A, *TEF1* gene, partial coding sequence	100%	99.86%	0.0
OP985475	Fc_V2	10	MG195126.1	*Fusarium culmorum* isolate Lapemi 16, *TEF1* gene, partial coding sequence	100%	100.00%	0.0
			LT548347.1	*Fusarium culmorum* strain IIE24, *TEF1* gene, partial coding sequence	100%	100.00%	0.0
			LT548337.1	*Fusarium culmorum* strain IIA28, *TEF1* gene, partial coding sequence	100%	100.00%	0.0
OP985476	Fc_S1	11	MG195126.1	*Fusarium culmorum* isolate Lapemi 16, *TEF1* gene, partial coding sequence	100%	100.00%	0.0
			LT548347.1	*Fusarium culmorum* strain IIE24, *TEF1* gene, partial coding sequence	100%	100.00%	0.0
			LT548337.1	*Fusarium culmorum*, strain IIA28, *TEF1* gene partial coding sequence	100%	100.00%	0.0
OP985477	Fc_At4	12	MG195126.1	*Fusarium culmorum* isolate Lapemi 16, *TEF1* gene, partial coding sequence	100%	100.00%	0.0
			LT548347.1	*Fusarium culmorum* strain IIE24, *TEF1* gene, partial coding sequence	100%	100.00%	0.0
			LT548337.1	*Fusarium culmorum* strain IIA28, *TEF1* gene, partial coding sequence	100%	100.00%	0.0
OP985478	Fc_At5	13	MG195126.1	*Fusarium culmorum* isolate Lapemi 16, *TEF1* gene, partial coding sequence	100%	100.00%	0.0
			LT548347.1	*Fusarium culmorum* strain IIE24, *TEF1* gene, partial coding sequence	100%	100.00%	0.0
			LT548337.1	*Fusarium culmorum* strain IIA24, *TEF1* gene, partial coding sequence	100%	100.00%	0.0
OP985479	Fg_At7	14	OP105199.1	*Fusarium graminearum* isolate JL10-1, *TEF1* gene, partial coding sequence	100%	100.00%	0.0
			MZ078991.1	*Fusarium graminearum* strain 5HS, *TEF*1 gene, partial coding sequence	100%	100.00%	0.0
			MT237780.1	*Fusarium graminearum* isolate YNF16-37, *TEF1* gene, partial coding sequence	100%	100.00%	0.0

OP985480	Fg_L7	15	OP105199.1	*Fusarium graminearum* isolate JL10-1, *TEF1* gene, partial coding sequence	100%	100.00%	0.0
			MZ078991.1	*Fusarium graminearum* strain 5HS, *TEF1* gene, partial coding sequence	100%	100.00%	0.0
			MT237780.1	*Fusarium graminearum* isolate YNF16-37, *TEF1* gene, partial coding sequence	100%	100.00%	0.0
OP985481	Fg_V1	16	OP105199.1	*Fusarium graminearum* isolate JL10-1, *TEF1* gene, partial coding sequence	100%	100.00%	0.0
			MZ078991.1	*Fusarium graminearum* strain 5HS, *TEF1* gene, partial coding sequence	100%	100.00%	0.0
			MT237780.1	*Fusarium graminearum* strain IF36, *TEF 1* gene, partial coding sequence	100%	100.00%	0.0
OP985482	Fg_L5	17	LT548448.1	*Fusarium graminearum* isolate JL10-1, *TEF1* gene, partial coding sequence	100%	100.00%	0.0
			OP105199.1	*Fusarium graminearum* isolate JL10-1, *TEF1* gene, partial coding sequence	100%	100.00%	0.0
			OK644316.1	*Fusarium graminearum, TEF1* gene, partial coding sequence	100%	100.00%	0.0
OP986483	Fg_Md3	18	LT548448.1	*Fusarium graminearum* isolate JL10-1, *TEF1* gene, partial coding sequence	100%	100.00%	0.0
			OP105199.1	*Fusarium graminearum* isolate JL10-1, *TEF1* gene, partial coding sequence	100%	100.00%	0.0
			OK644316.1	*Fusarium graminearum, TEF1* gene, partial coding sequence	100%	100.00%	0.0
OP985484	Fg_V7	19	LT548448.1	*Fusarium graminearum* isolate JL10-1, *TEF1* gene, partial coding sequence	100%	100.00%	0.0
			OP105199.1	*Fusarium graminearum* isolate JL10-1, *TEF1* gene, partial coding sequence	100%	100.00%	0.0
			OK644316.1	*Fusarium graminearum, TEF1* gene, partial coding sequence	100%	100.00%	0.0

Table S2. BLASTn results for RNA polymerase second largest subunit (*RPB2*) gene sequences of each of the 19 *Fusarium* isolates. For each compared sequence top three highly similar (with the highest percentage of identity) results are provided.

Accession number	Isolate name	Isolate number	Top three results from BLASTn				
			Accession number	Accession description	Query cover	Percent identity	E-value
	Fr_S1	1	MW832205.1	*Fusarium redolens* strain BMU 03123, *RPB2* gene, partial coding sequence	100%	100.00%	0.0
			MW091315.1	*Fusarium redolens* isolate NA_FRA01, *RPB2* gene, partial coding sequence	100%	100.00%	0.0
			GU250699.1	*Fusarium redolens* strain CBS:743.97, *RPB2* gene, complete coding sequence	95%	100.00%	0.0
	Fo_Mv1	2	MN457541.1	*Fusarium oxysporum* voucher VPRI42888, *RPB2* gene, partial coding sequence	100%	100.00%	0.0
			MN457539.1	*Fusarium oxysporum* voucher VPRI42760, *RPB2* gene, partial coding sequence	100%	100.00%	0.0
			MN457534.1	*Fusarium oxysporum* voucher VPRI42252, *RPB2* gene, partial coding sequence	100%	100.00%	0.0
	Fo_At1	3	MN457541.1	*Fusarium oxysporum* voucher VPRI42888, *RPB2* gene, partial coding sequence	100%	100.00%	0.0
			MN457539.1	*Fusarium oxysporum* voucher VPRI42760, *RPB2* gene, partial coding sequence	100%	100.00%	0.0
			MN457534.1	*Fusarium oxysporum* voucher VPRI42252, *RPB2* gene, partial coding sequence	100%	100.00%	0.0
	Fo_Mv3	4	MN457491.1	*Fusarium oxysporum* voucher RGB6505, *RPB2* gene, partial coding sequence	100%	100.00%	0.0
			MN457542.1	*Fusarium oxysporum* voucher VPRI42889, *RPB2* gene, partial coding sequence	100%	99.69%	0.0
			MN457532.1	*Fusarium oxysporum* voucher VPRI42190, *RPB2* gene, partial coding sequence	100%	99.69%	0.0
	Fo_L1	5	MN457541.1	*Fusarium oxysporum* voucher VPRI42888, *RPB2* gene, partial coding sequence	100%	100.00%	0.0
			MN457539.1	*Fusarium oxysporum* voucher VPRI42760, *RPB2* gene, partial coding sequence	100%	100.00%	0.0
			MN457534.1	*Fusarium oxysporum* voucher VPRI42252, *RPB2* gene, partial coding sequence	100%	100.00%	0.0
	Fo_V3	6	MN457491.1	*Fusarium oxysporum* voucher RG6505, *RPB2* gene, partial coding sequence	100%	100.00%	0.0
			MN457542.1	*Fusarium oxysporum* voucher VPRI42889, *RPB2* gene, partial coding sequence	100%	99.69%	0.0
			MN457532.1	*Fusarium oxysporum* voucher VPRI42190, *RPB2* gene, partial coding sequence	100%	99.69%	0.0
	Fo_V1	7	MN457541.1	*Fusarium oxysporum* voucher VPRI42888, *RPB2* gene, partial coding sequence	100%	100.00%	0.0
			MN457539.1	*Fusarium oxysporum* voucher VPRI42760, *RPB2* gene, partial coding sequence	100%	100.00%	0.0

		MN457534.1	*Fusarium oxysporum* voucher VPRI42252, *RPB2* gene, partial coding sequence	100%	100.00%	0.0
Fo_Md7	8	MN457541.1	*Fusarium oxysporum* voucher VPRI42888, *RPB2* gene, partial coding sequence	100%	100.00%	0.0
		MN457539.1	*Fusarium oxysporum* voucher VPRI42760, *RPB2* gene, partial coding sequence	100%	100.00%	0.0
		MN457534.1	*Fusarium oxysporum* voucher VPRI42252, *RPB2* gene, partial coding sequence	100%	100.00%	0.0
Fo_An7	9	MN457541.1	*Fusarium oxysporum* voucher VPRI42888, *RPB2* gene, partial coding sequence	100%	100.00%	0.0
		MN457539.1	*Fusarium oxysporum* voucher VPRI42760, *RPB2* gene, partial coding sequence	100%	100.00%	0.0
		MN457534.1	*Fusarium oxysporum* voucher VPRI42252, *RPB2* gene, partial coding sequence	100%	100.00%	0.0
Fc_V2	10	GQ915490.1	*Fusarium culmorum* strain FRC R-09618, *RPB2* gene, partial coding sequence	100%	100.00%	0.0
		MT997139.1	*Fusarium culmorum* isolate M62A, *RPB2* gene, partial coding sequence	98%	100.00%	0.0
		MH582079.1	*Fusarium culmorum* strain MRC 1371, *RPB2* gene, partial coding sequence	98%	100.00%	0.0
Fc_S1	11	GQ915490.1	*Fusarium culmorum* strain FRC R-09618, *RPB2* gene, partial coding sequence	100%	100.00%	0.0
		MT997139.1	*Fusarium culmorum* isolate M62A, *RPB2* gene, partial coding sequence	100%	100.00%	0.0
		MH582079.1	*Fusarium culmorum* strain MRC 1371, *RPB2* gene, partial coding sequence	98%	100.00%	0.0
Fc_At4	12	GQ915490.1	*Fusarium culmorum* strain FRC R-09618, *RPB2* gene, partial coding sequence	100%	100.00%	0.0
		MT997139.1	*Fusarium culmorum* isolate M62A, *RPB2* gene, partial coding sequence	98%	100.00%	0.0
		MH582079.1	*Fusarium culmorum* strain MRC 1371, *RPB2* gene, partial coding sequence	98%	100.00%	0.0
Fc_At5	13	GQ915490.1	*Fusarium culmorum* strain FRC R-09618, *RPB2* gene, partial coding sequence	100%	100.00%	0.0
		MT997139.1	*Fusarium culmorum* isolate M62A, *RPB2* gene, partial coding sequence	98%	100.00%	0.0
		MH582079.1	*Fusarium culmorum* strain FRC R-09618, *RPB2* gene, partial coding sequence	98%	100.00%	0.0
Fg_At7	14	MH582179.1	*Fusarium graminearum* strain MRC 2563, *RPB2* gene, partial coding sequence	98%	100.00%	0.0
		KT855207.1	*Fusarium graminearum* strain CBS 123688, *RPB2* gene, partial coding sequence	94%	100.00%	0.0
		MN892317.1	*Fusarium graminearum* strain A2s4, *RPB2* gene, partial coding sequence	98%	100.00%	0.0
Fg_L7	15	MH582179.1	*Fusarium graminearum* strain MRC 2563, *RPB2* gene, partial coding sequence	98%	100.00%	0.0
		KT855207.1	*Fusarium graminearum* strain CBS 123688, *RPB2* gene, partial coding sequence	94%	100.00%	0.0

		MN892317.1	*Fusarium graminearum* strain A2s4, *RPB2* gene, partial coding sequence	98%	100.00%	0.0
Fg_V1	16	MH582179.1	*Fusarium graminearum* strain MRC 2563, *RPB2* gene, partial coding sequence	98%	100.00%	0.0
		KT855207.1	*Fusarium graminearum* strain CBS 123688, *RPB2* gene, partial coding sequence	94%	100.00%	0.0
		MN892317.1	*Fusarium graminearum* strain A2s4, *RPB2* gene, partial coding sequence	98%	100.00%	0.0
Fg_L5	17	MH582179.1	*Fusarium graminearum* strain MRC 2563, *RPB2* gene, partial coding sequence	98%	100.00%	0.0
		KT855207.1	*Fusarium graminearum* strain CBS 123688, *RPB2* gene, partial coding sequence	94%	100.00%	0.0
		MN892317.1	*Fusarium graminearum* strain A2s4, *RPB2* gene, partial coding sequence	98%	100.00%	0.0
Fg_Md3	18	MH582179.1	*Fusarium graminearum* strain MRC 2563, *RPB2* gene, partial coding sequence	98%	100.00%	0.0
		KT855207.1	*Fusarium graminearum* strain CBS 123688, *RPB2* gene, partial coding sequence	94%	100.00%	0.0
		MN892317.1	*Fusarium graminearum* strain A2s4, *RPB2* gene, partial coding sequence	98%	99.79%	0.0
Fg_V7	19	MH582179.1	*Fusarium graminearum* strain MRC 2563, *RPB2* gene, partial coding sequence	98%	100.00%	0.0
		KT855207.1	*Fusarium graminearum* strain CBS 123688, *RPB2* gene, partial coding sequence	94%	100.00%	0.0
		MN892317.1	*Fusarium graminearum* strain A2s4, *RPB2* gene, partial coding sequence	98%	99.79%	0.0

Table S3. Details on the origin of the reference *Fusarium* spp. isolates (including the species, isolate name, source and country of isolation) and the GenBank accession numbers of their *TEF1* and *RPB2* sequences included in phylogenetic analysis (see Figure 2).

Species	Strain/Isolate name	Isolation source	Country of isolation	GenBank Accession Number	
				TEF1	***RPB2***
Fusarium culmorum	NRRL 25475	barley	Denmark	KY873384	MW233425
Fusarium culmorum	MRC 1787	unknown	Canada	MH582254	MH582106
Fusarium culmorum	MRC 2197	potato	Germany	MH582257	MH582139
Fusarium culmorum	MRC 1890	barley	Germany	MH582256	MH582109
Fusarium culmorum	MRC939	corn	Australia	MH582258	MH582222
Fusarium culmorum	MRC 1889	barley	Germany	MH582255	MH582108
Fusarium culmorum	M62A	wood	China	MT661593	MT997139
Fusarium culmorum	CBS 139512	weat	Poland	KT855186	KT855212
Fusarium graminearum	CBS 123688	oat	Sweden	KT855181	KT855207
Fusarium graminearum	MRC 3082	feed	Germany	MH 582244	MH582207
Fusarium graminearum	A2s4	soybean	China	MK560307	MN892317
Fusarium graminearum	A1s4	soybean	China	MK560306	MN892318
Fusarium graminearum	NL19-100008	soil	The Netherlands	MZ921906	MZ921775
Fusarium graminearum	MRC 3081	feed	Germany	MH582243	MH582206
Fusarium graminearum	MRC 2940	millet	Hungary	MH582242	MH582205
Fusarium graminearum	CBS 136009	barley	Germany	MW928838	MW928826
Fusarium graminearum	NRRL 31084	maize	USA	MW233103	MW233447
Fusarium graminearum	SYS 145	maize	China	OM985204	OP435990
Fusarium graminearum	SYS 144	maize	China	OM985203	OP435989
Fusarium oxysporum	B3s6	soybean	China	MK560296	MN892310
Fusarium oxysporum	NRRL 62545	human	USA	KR673926	KR674016
Fusarium oxysporum	NRRL 54996	pinguin	USA	KC808227	KC808366
Fusarium oxysporum	NRRL 62547	dog	USA	KC808224	KC808367
Fusarium oxysporum	NRRL 25387	human	USA	HM347117	HM347209
Fusarium oxysporum	B5s2	soybean	China	MK560297	MN892309
Fusarium oxysporum	B2s3	soybean	China	MK560294	MN892312
Fusarium oxysporum	B2s1	soybean	China	MK560293	MN892313
Fusarium oxysporum	A7r1	soybean	China	MK560302	MN892305
Fusarium oxysporum	A6s3	soybean	China	MK560301	MN892306
Fusarium redolens	NRRL 25600	needle grass	Tunisia	MT409453	MT409443
Fusarium redolens	NRRL 22901	Needle grass	Tunisia	MT409452	MT409442
Fusarium redolens	NRRL 54967	cat	USA	KC808221	KC808363
Fusarium solani	NRRL 43474	human	USA	EF452945	EF469984
Fusarium solani	GJS 09-1466 T	potato	Slovenia	KT313611	KT313623
